# Studies on Colchicine Induced Chromosome Doubling for Enhancement of Quality Traits in Ornamental Plants

**DOI:** 10.3390/plants8070194

**Published:** 2019-06-28

**Authors:** Ayesha Manzoor, Touqeer Ahmad, Muhammad Ajmal Bashir, Ishfaq Ahmad Hafiz, Cristian Silvestri

**Affiliations:** 1 Barani Agricultural Research Institute, Chakwal 48800, Pakistan; 2 Department of Horticulture, PMAS Arid Agriculture University, Rawalpindi, Punjab 46000, Pakistan; 3 Department of Agriculture and Forest Sciences (DAFNE), University of Tuscia, 01100 Viterbo, Italy

**Keywords:** chromosomes, colchicine, diploid, induction, ornamental, polyploidy, tetraploid

## Abstract

Polyploidy has the utmost importance in horticulture for the development of new ornamental varieties with desirable morphological traits referring to plant size and vigor, leaf thickness, larger flowers with thicker petals, intense color of leaves and flowers, long lasting flowers, compactness, dwarfness and restored fertility. Polyploidy may occur naturally due to the formation of unreduced gametes or can be artificially induced by doubling the number of chromosomes in somatic cells. Usually, natural polyploid plants are unavailable, so polyploidy is induced synthetically with the help of mitotic inhibitors. Colchicine is a widely used mitotic inhibitor for the induction of polyploidy in plants during their cell division by inhibiting the chromosome segregation. Different plant organs like seeds, apical meristems, flower buds, and roots can be used to induce polyploidy through many application methods such as dipping/soaking, dropping or cotton wool. Flow cytometry and chromosome counting, with an observation of morphological and physiological traits are routine procedures for the determination of ploidy level in plants.

## 1. Introduction

Polyploidy was first discovered in 1907 and was thought to be responsible for increasing the number of genomic copies that are heritable [1]. Polyploidy is the condition of having more than two sets of chromosomes [2] and also acts as an important mechanism for plant evolution. Paleopolyploidy is commonly practiced in plants and is the most important feature in the evolutionary history of all land plants [3]. It has been observed that approximately every plant has somehow experienced polyploidy at specific point during their evolution. However, most plants have experienced recent chromosome duplication, known as neopolyploidy. Almost 70% of flowering plants (angiosperms) have undergone polyploidy several times during their evolution [4]. Polyploidy leads to changes in gene dosage that causes chromosomal rearrangements, epigenetic remodeling and reunion of divergent gene regulatory hierarchies. These changes may bring out novel variations or may result in speciation and reproductive isolation [5]. Polyploids may develop naturally by cytological mechanisms like production of unreduced gametes [6] or may also occur due to unfavorable environmental conditions [1].

Polyploidy can be autopolyploid, which involves the chromosome duplication of same ancestors or it can be allopolyploid, when the affected plants are produced during the first hybridization of parents with different genomic content and then duplication of this hybridized genomic material occurs. Plant breeders select polyploid plants in order to improve their agronomic traits, some of which were developed by autopolyploidisation (within the species) or by allopolyploidisation of different species (interspecific hybrids) [7]. Compared to allopolyploids that are more common in nature, autopolyploids pose a great advantage as these organisms maximize the number of genes and allele variants. Some traits like drought tolerance, pest resistance, blooming time, size of organ, apomixis and biomass increase the chances of polyploid plants being selected for agriculture or to enter new niches [8]. The ability of polyploid plants to establish themselves in a wide range of habitats and to survive in adverse environmental conditions makes them successful against their diploid ancestors, due to the presence of additional alleles that increase their heterozygosity [1].

Phenotypic and genetic changes in plants caused by polyploidy may occur due to an increase in cell size, allelic diversity (level of heterozygosity), gene silencing and gene dosage effect or because of epigenetic and genetic interactions. Unreduced (*2n*) gametes having somatic chromosome numbers are the main factors for polyploidy induction in nature. In plant breeding, the use of *2n* gametes results in production of meiotic polyploids that are very helpful for crop improvement as they combine the genetic effects of different plants of increased ploidy level and meiotic recombination as well. Unreduced gametes transmit genetic diversity efficiently in a form of both quantitative and qualitative traits in cultivated plants and are considered as effective alternatives for somatic (mitotic) chromosome doubling [9].

Polyploidy increases genetic base through the development of breeding lines in a short time span and restoring hybrid fertility in interspecific and interploidy crosses [6]. There are three noticeable evident advantages of polyploidy comprising of heterosis, gene redundancy and asexual reproduction. Heterosis results in vigorous polyploid plants as compared to their diploid parents whereas gene redundancy protects the polyploid plants from the deleterious effects of mutation. Gene redundancy also causes a buffering effect in which extra copies of *wild type* alleles conceive the effect of deleterious alleles. In this way, extra copies of genes provide a protection against inbreeding depression and single locus deleterious mutation effects. Another important benefit of gene redundancy is found in terms of duplicated gene pairs, in which a member mutates and obtains certain novel function without affecting the essential functions [10]. Asexual reproduction allows the polyploid plants to reproduce even in the absence of their sexual mates [11].

## 2. Breeding of Ornamental Crops

For breeding of ornamental crops, particularly those species in which very little breeding has been done, various biotechnological interventions like polyploidization, haploids, mutation breeding and in vitro somaclonal variations may speed up breeding and selection of novel mutants. Among all these methods, mutation breeding and polyploidy are most likely being used in asexual propagated crops to develop novel varieties and to achieve different genetic variations. In fact, the mutation effect is easily visible in ornamentals, in terms of change in flower color, shape and size [12]. Polyploids have been successfully produced during the last few decades in many ornamental crops [13]. Polyploidy breeding is an efficient method as compared to mutation breeding and conventional cross breeding due to easy handling within a short span of time which increases the germplasm availability as well [14]. Moreover, artificial polyploidization, in contrast to mutation breeding which involves gene mutation, results in whole genome alteration that produces greater phenotypic variations as compared to single gene mutation [15]. 

An increase in cell size is the most important result of polyploidy that occurs due to the addition of extra gene copies. This effect of polyploidy is known as “gigas effect” [10]. Polyploidy causes intensification of flower color, increasing flower size and alters the plant shape [13]. Along with increasing the size of various vegetative and reproductive parts in tetraploid plants, chromosome duplication may also alter the plant growth habits, sexuality patterns, sterility and sometimes, increases the cold hardiness [16]. Due to the increase sterility of induced tetraploids, vegetative propagation of ornamentals remains the major propagation system and it prevents the flower contamination with foreign pollens [17]. A delay in flowering, with increase in flower diameter and malformation of flowers along with floral parts of different colors on the same branch, are the common characteristics of colchicine treated plants [18].

## 3. Induction of Polyploidy

**Figure 1:**
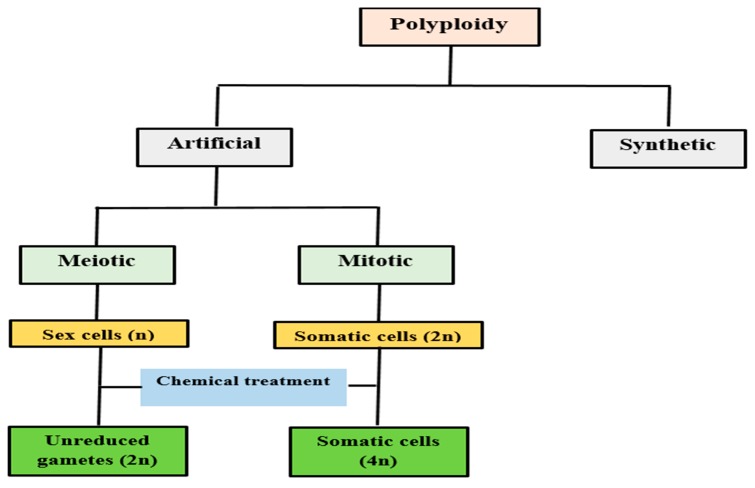
Systematic diagram of polyploidy induction through artificial means.

Various vegetatively propagated crops are polyploids. However, polyploidy does not exist naturally in all plant genera, so it has been artificially induced in many economically important crops over last few decades [19]. Formation of induced polyploids is an effective way to develop genetic variation for study of genetics and plant breeding [20]. Polyploidy can be artificially induced through interspecific hybridization, in vitro endosperm culture or somatic cell doubling through colchicine [21]. There are two different ways to induce polyploidy artificially: (i) meiotic (sexual) or (ii) mitotic (somatic). In meiotic polyploidization, *2n* pollens (gametes) are produced (Figure 1). Naturally produced sexual polyploids, particularly triploids produced through formation of *2n* gametes, have been studied as important features in the evolution of flowering plants. Although, artificially induced sexual polyploids are less frequently used for breeding purpose. In mitotic polyploidization, the duplication of chromosome is done in somatic cells. This type of chromosome duplication is widely used to induce tetraploids through anti-mitotic chemicals [22]. However, chromosome duplication of somatic cells form additional copies of chromosome and existing genes but many variations occur after the chromosome doubling which leads to variations in plant phenotypes [20]. However, in many crops, a large number polyploid plants have been induced. They do not usually exhibit higher yield as compared to their diploid parents. This may be due to improvements occurring in that plant’s organs which are not of economic interest. Each crop species has different responses towards polyploidization, which depends on their genomic structure, reproduction patterns, ancestral ploidy level and the purpose for which the crop has been cultivated [10].

Different plant organs/tissues such as roots, seeds, flower axillary and apical meristems are currently being used for polyploidy induction [23]. It is effective to induce polyploidy in tissues that are obtained from those organs which have highly morphogenetic responses such as embryo, apical meristems and young inflorescence [24]. Although colchicine is readily used to induce polyploidy in many crops, there are still limitations in developing effective protocols for polyploidy induction and for the production of high yielding ploidy mutant [25].

### 3.1. Colchicine Induced Polyploidy/Mutation

The importance of polyploidy in plant breeding appeared to be of great interest when the mitotic inhibitor (colchicine) was first discovered in 1930s [26]. Colchicine has been used for treatment of gout disease since 1810. Colchicine is extracted from bulbs (0.1–0.5%) and seeds (0.2%–0.8%) of autumn crocus or meadow saffron (*Colchicum autumnale*) and possesses an extremely poisonous alkaloid character. It is readily soluble in cold water, chloroform or alcohol but it has low solubility in hot water [27]. Colchicine is an important mutagen that works by preventing the microtubules formation and doubles the number of chromosomes. It is commonly used to develop polyploid plants and functions as a mitotic poison by producing many mutagenic effects on plants [28]. As microtubules function in chromosome segregation, colchicine induces polyploidy by preventing the segregation of chromosomes during meiosis that results into half of the gametes (sex cells) containing double the chromosome number than usual. Whereas, half of the gametes do not contain any chromosomes and produce embryos with doubled chromosome numbers [29]. Colchicine not only helps in the doubling of chromosomes, but it also induces mutation in plants. Plants that have been mutated through colchicine are known as colchi-mutants [12]. According to the literature, a wide range of colchicine concentrations have been used for the induction of polyploidy in different plant species such as the lowest concentration of 0.00001% in campion (*Lychnic senno*) to the extremely high concentration of 1.5% in Maule’s quince (*Chaenomeles japonica*), and have successfully induced polyploidy [30]. However, colchicine usually has low affinity to tubulins in plant cells, thus higher concentrations are used for effective results [15].

Colchicine is commonly applied in a form of an aqueous solution; however, it is unstable in water. Therefore, it is advisable to make a fresh aqueous solution before treatment [27]. Colchicine concentrations for seed treatment usually range from 0.1%–0.8%, but high doses cause malformation and reduce the production of tetraploids plants. Thus, it is advisable to use colchicine at concentrations that are as low as possible [31]. As colchicine is highly toxic to plants, therefore, low doses with prolonged exposure period are considered reliable to reduce its toxic effect and increase the polyploids production rate [13].

### 3.2. Methods of Application

Among different application methods, change in ploidy level of plants through chemicals has been proven to be more effective. Many chemicals work as inhibitors to block the progress of cell division cycle (mitosis) in plant cells, like colchicine that is used to block metaphase of cell cycle [13]. Methods of anti-mitotic application depend upon the plant type. One of the simplest, easiest and effective methods is to use a large number of seedlings that have small and actively growing meristematic tissues. Seedlings can be dipped/soaked or apical meristems can be submerged in anti-mitotic agent solution of different concentrations at different exposure times or frequencies. Shoots of older plants can also be used for polyploidy induction, but this method is not successful as it may yield large number of cytochimeras. However, treatment of sub-axillary and small axillary meristematic tissues is considered to be effective, whilst, on the other hand, growing buds can also be treated with chromosome doubling agents with the help of cotton, lanolin and agar or by dipping branch tips in solutions of chemicals. Wetting agents and surfactants are also sometimes used to enhance the penetration of a chemical [32]. The most successful method for tetraploidy induction is through seed treatment. Colchicine treatment of pre-germinated seeds having emerging roots is effective as compared to dry or germinated seeds because large numbers of tetraploid plants are produced through it [31,33].

### 3.3. Confirmation

For crop breeding, it is important to identify the correct ploidy level at various growth stages through quick, simple and easy methods [34]. Identification or determination of correct ploidy level is very important in breeding of polyploid and asexually propagated crops as they might have several polyploid series or chimeras in their tissues [7]. For confirmation of polyploidy, different direct and indirect methods have been used. Indirect methods are easy, less time consuming and use simple instruments for screening [15]. Different morphological and physiological traits, particularly pollen diameter, number of chloroplast, stomatal size and stomatal density can be studied through these indirect methods [35]. In various species, there is a direct relationship between plant ploidy level and different physiological characteristics like stomatal length, width, density, pollen grain diameter and number of chloroplasts in guard cells. However, some disadvantages like environmental sensitivity, need for calibration or identification of mixoploids and chimeras (rather than polyploids) limit the use of these traits to identify correct ploidy level. In direct methods, techniques like chromosome counting have been examined as an effective and reliable method, but it is a laborious process, particularly for plant species with highly dense cytoplasm consisting of a large number of chromosomes [36]. Moreover, a specific protocol is required for each plant species. Thus, flow cytometry is considered to be a more reliable, rapid and simple method to analyze a large number of samples in a very short time period [10]. Flow cytometry is generally used to quantify DNA in nuclei. Quantity of DNA can be determined directly from the leaf tissues with this method. This method is also suitable for those plant species in which root tip studies are not effective due to small size and ineffective spreading of chromosome [37]. Furthermore, this method can also evaluate large number of nuclei (100–10,000 cells per second) from different cell layers and types of tissues [15].

### 3.4. Chimeras

It is important to identify the cytochimeras (plants whose ploidy level differ in different types of tissues) when polyploids are used for breeding purpose. Meristematic cells are usually divided into three histogenic layers LI, LII and LIII. Antimitotic agents might increase ploidy level in all three layers or in one or two of them. Thus, to study the reproductive behavior, it is necessary to identify the ploidy level of cortical layer or LII layer, that can be measured through pollen grain diameter and by meiotic studies of reproductive tissues (anthers). For L1 and LIII layer, guard cell studies and chromosome counting can accurately determine the ploidy level, respectively [32]. Occurrence of chimeras instead of stable tetraploid plants shows that all cells present in histogenic layers (LI, LII and LIII) of meristems tissues are not treated at the same time. The successful conversion of diploid plants into stable polyploids depends on the balance of colchicine concentration and exposure time that double the genomic content of each cell of treated tissues and ensuring that the survival and growth and development of induced plants is not severely affected [38].

### 3.5. Improvement of Ornamentals by Polyploidization

Polyploid plants are noticeably different or sometimes superior from diploid ones in terms of their phenotypic expression, which includes morphological, physiological, biochemical and cellular changes. These polyploid plants can also be taken as a new variation or a genotype that can be used in future breeding programs for crop improvement. Due to the immense importance of polyploidy, it has been artificially induced in many economically important crops, however the majority of success has been reported in ornamental industry. Chromosome doubling through colchicine by using different application methods has been obtained in many ornamental crops such as lily, salvia, phlox, gladiolus, petunia and marigold (Table 1, Table 2, Table 3 and Table 4). 

Polyploidy causes a wide range of effects on plants, but these effects depend upon species and range of different ploidy level within a same species, degree of heterozygosity and rely on various mechanisms that are related to gene dose effects, gene silencing, regulation of specific traits and gene interaction [32].

### 3.6. Morphology

A large number of morphological effects have been obtained from induced polyploidization. However, one of the immediate effects of polyploidy is an increase in cell size due to the increase in nuclear content which causes a reduction in a cell division during their growth and development. This “gigas effect” is mostly observed in different plant organs of commercial interests such as leaves, seeds and flowers [79]. Doubling through colchicine caused an increase in number of leaves, number of branches, plant height and stem length in salvia (*Salvia coccinea* cv Coral Nymph) [42], jasmine tobacco (*Nicotiana alata*) [43], selfheal (*Prunella vulgaris*) [48], lily (*Lilium*) [41], chaste tree (*Vitex agnus castus*) [12], orchid (*Dendrobium nobile*) [40], ornamental ginger [80], crape myrtle (*Lagerstroemia indica*) [62], calendula (*Calendula officinalis*) [28], matted sea-lavender (*Limonium bellidifolium*) [53], white orchid tree (*Bauhinia acuminate*) [55] and London plane (*Planatus acerifolius*) [71]. Induced polyploidy also increased the leaf color in balsam (*Impatiens balsamina*), [73], self-heal (*Prunella vulgaris*) [48], wishbone flower (*Torenia fournieri*) [46], marigold (*Tagates erecta*) [63], chaste tree (*Vitex agnus castus*) [12] and chrysanthemum (*Dendranthema grandiflora*) [78], along with increasing their leaf area as well. Induced polyploidy produced ovate shaped leaves having obtuse base in gymnostachyum (*Gymnostachyum zeylanicum*) [39] and whorled arrangement of leaves in balloon flower (*Platycodon grandiflorus*) [67]. Highest variation in color and shape of leaves has been observed in tetraploid plants of pelargonium (*Pelargonium graveolens*), [72]. 

Mitotic chromosome doubling through colchicine treatment results in production of large sized inflorescence with increased floral parts in salvia (*Salvia coccinea* cv. Coral Nymph), however flowering has been delayed up to 10–30 days [42]. In chaste tree (*Vitex agnus castus*), polyploid plants had larger flowers with longest posterior petals and unique colors. Tetraploid plants of feverfew (*Tanacetum parthenium*) had increased flower weight and diameter but produced only up to 50% of flowering as compared to its diploid plants [44]. Also, in ornamental wild ginger species (*Larsenianthus* careyanus), chromosome doubling caused an increase in leaf number, lamina length, flower size along with length of inflorescence and spike length [80]. Dense flowering with erect, compacted and short inflorescence was produced in gymnostachyum (*Gymnostachyum zeylanicum*), from 0.04% colchicine treated plants [39]. Tetraploidization in African violets (*Saintpaulia ionantha*) produced 5% white flowers with purple margin. After 7 days of flowering, flower color pattern changed from white petals with purple border to whole purple petals and this pattern has been maintained for six successive generations [60]. Tetraploid plants of pelargonium (*Pelargonium graveolens*) cv. Black Velvet Scarlet F1 produced flowers with rough edges, where flowers of cv. Gizela produced burnt margins [72]. Similarly, colchicine treatment produced more flowering with large stigma in jasmine tobacco (*Nicotiana alata*) [43], larger flower height with increased lip width in wishbone flower (*Torenia fournieri)* [46] increased the number of petals in garden balsam (*Impatiens balsamina*) [73] and increased flower diameter up to 1.2–1.3 folds in tetraploid plants of matted sea-lavender (*Limonium bellidifolium*) as compared to its diploid plants [53]. Colchi-tetraploids of chrysanthemum (*Chrysanthemum carinatum*) had larger flowers with thicker petals that helped to improve their vase life [66]. Also, in gladiolus, colchicine induced putative polyploids had larger flower size with maximum vase life. Moreover, novel variation in flower morphology like serrated margins with ruffled edges along with pointed outgrowth has been observed in flower petals of gladiolus [57]. Higher colchicine concentration in African marigold (*Tagetes erecta*) initiated early flowering (59 days) in treated plants as compared to control plants (80 days). Also, a higher number of flowers with increased diameter and weight of flowers have been produced in polyploid plants [81].

Apart from improving ornamental traits like flower or leaves in ornamental plants, polyploidy also increases the plants yield in the form of both sexual and asexual reproductive structures. After colchicine treatment, a significant increase in seed size and weight was observed in crape myrtle (*Lagerstroemia indica*) [62] and Madgascar periwinkle (*Catharanthus roseus*) [74]. Whereas, colchicine also increased seed number, seed weight and fruit setting percentage in balsam (*Impatiens balsamina*) [59]. Similarly, in vegetatively propagated crops like *Lilium,* induced chromosome doubling produced wider bulb scale [41], however, in orchid (*Dendrobium nobile*), polyploidization decreased pseudobulb diameter up to 64.9% [50]. 

### 3.7. Physiology

In addition to the obvious alterations in the morphology of ornamental plants, polyploidy can also show a significant impact on the number of plant physiological processes such as water relations. Larger sized stomata with lower frequency per unit area have been observed in polyploid plants of feverfew (*Tanacetum parthenium*), phlox (*Phlox drummondi)* [44,45], salvia (*Salvia hains*) [47], petunia (*Petunia hybrida*) [61], African marigold (*Tagates erecta*) [63], chrysanthemum (*Dendranthema indicum* var. Aromaticum) [64] and celosia (*Celosia argentea*) [82]. It is illustrated in the results that these changes reduced the transpiration rate (overall gaseous exchange rate) in polyploid plants. Additionally, increases in vacuole size and thicker leaves, also allows for retaining higher water content, which can be utilized during drought period conditions. Therefore, these polyploid plants could be grown in water limited areas and can also be bred with other species in order to develop drought tolerant genotypes. This approach could be helpful for domestication of some species into warmer and hot climate. 

### 3.8. Resorting Fertility in Wide Hybrids

Hybrids between different genera or species are usually sterile due to the failure of chromosome pairing under meiosis. By chromosome doubling of wide hybrid, each chromosome gets its exact copy and chromosomal homology, thus its fertility is restored. This technique has been used to restore fertility in lavandin (*Lavandula* × *intermedia*), which is grown for both oil and ornamental purpose. Along with restoration of fertility, heavier seeds have been produced in offspring with respect to their parent seed weight. Similarly, in spurflowers (South African *Plectranthus*), polyploidy has been induced in F_1_ sterile diploid hybrids (2*n* = 28) in order to obtain triploid crosses (4*n* × 2*n*) [83].

### 3.9. Overcoming Hybridization Barrier

Desirable crosses are sometimes difficult to obtain due to different ploidy level of parents. This type of interploid barrier usually occurs due to imbalance or abnormal endosperm formation and the seeds can be developed normally with a ratio of 2:1 (maternal and paternal) in the genomic makeup of the endosperms for two diploid parents. The seed that does not meet the normal development are generally aborted or may be underdeveloped. In order to break these barriers in hybridization, ploidy levels of one or both parents are manipulated to match before the hybridization [84]. Chrysanthemum, *Dendranthema indicum* var. Aromaticum is diploid (*2n=2x=*18) and *Dendranthema* × *grandiflora* is polyploid (*2n=6x=54*). Thus, to create a new scented chrysanthemum, chromosome doubling has been induced through colchicine in *D. indicum* var. Aromaticum, in order to increase the chance of cross between both the parents [64].

### 3.10. Pest Resistance and Stress Tolerance

The effect of polyploidy on various biotic and abiotic stress tolerance and adaptability to a particular environment has been studied in many crops. Polyploid plants could have a better nutrient uptake, cold tolerance and improved resistance to insect/pest and pathogens. There are a number of ways to induce polyploidy in plants that can increase their adaptability and resistance against biotic and abiotic stresses. It can also be achieved by increasing the nuclear content that ultimately increases the gene expression which results in increased production of secondary metabolites. These metabolites including chemical defenses not only enhance the plant resistance and tolerance mechanisms, but they are also valuable from a pharmaceutical point of view. They can also be used to create allopolyploids among parents having diverse endogenous secondary metabolites as compared to their parent plants. Induced triploids and tetraploids plants of swamp rosemallow (*Hibiscus moscheutos*) showed resistance to aerial *Phytophthora* disease as compared to their diploid counterparts which were severely infected [54]. Also, in garden impatiens (*Impatiens walleriana*), synthetically produced tetraploids exhibited improved resistance to downy mildew [56]. 

There are several plants that can be used for both ornamental as well as medicinal purposes such as the celosia flower (*Celosia argentea*), which showed an increase in biomass and pharmaceutical compounds (alkaloid, phenols, anthocyanin) in tetraploid plants [82]. Similarly, in opium poppy (*Papaver somniferum*), a 25–50% increase in alkaloid contents was observed in colchicine treated plants [8]. Whereas, increased parthenolide content was observed in polyploid plants of feverfew (*Tanacetum parthenium*) [44]. 

## 4. Disadvantages

Apart from the major advantages, there are still several disadvantages that can occur due to the increase in chromosome number. Increase in nuclear content of cell causes an increase in cell volume. It is expected that the doubling of the cell genome will double the nucleus volume, but it only increases up to 1.6 fold in the nuclear envelope surface, which can disrupt the balance between the chromosome and nuclear components. This imbalance can cause various abnormalities during mitosis and meiosis [11,84,85]. It can also affect cell physiology (metabolism), regulation of gene expression and gene stability [86]. 

High levels of polyploidy, for instance in octoploids, can cause stunted and malformed phenotypes due to somatic instability and extreme gene redundancy, which leads to production of chimeric tissues [19]. As in chaste tree (*Vitex agnus castus*), colchicine showed its deleterious effects by producing dwarf tetraploid plants with short internodes and leaves of smaller size without formation of flower buds or flowers [12]. Similarly, scabbing and thickening of leaf tissue, leaf cupping, deformities, stunting and death of growing points also occurred in two cultivars of zinnia (*Zinnia violacea*) due to application of colchicine [75]. Pelargonium (*Pelargonium graveolens*), hexaploids and octoploids exhibited failure in blooming, and mixploid showed color loss of leaves [72]. In a study conducted in swamp rose-mallow (*Hibiscus moscheutos*), the flowers of colchicine induced tetraploid plants did not produce any pollens, whereas in triploid plants, non-viable pollen grains were produced but the fruit were aborted after pollination, thus leading to infertility of triploids [54].

**Figure 2:**
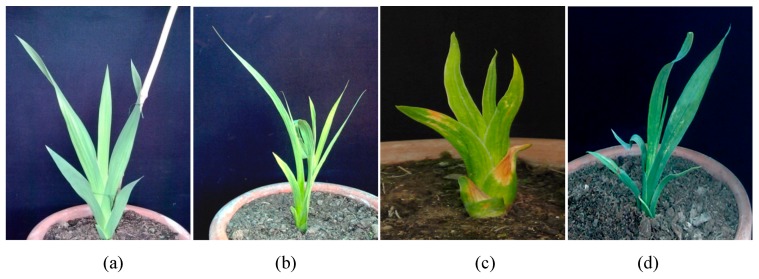
Production of foliar abnormalities in gladiolus cv. White Prosperity at different concentrations of colchicine: normal shaped leaves in control (**a**) but different abnormal leaves shapes observed in 0.1% (**b**), 0.2% (**c**) and 0.3% colchicine (**d**).

In another study conducted by Manzoor et al. [87] with regards to doubling tetraploid gladiolus chromosomes with the help of colchicine treatment, several negative effects of polyploidy were observed during different stages of growth and development. When colchicine treated gladiolus plants reached maturity, different types of foliar abnormalities were reported. As in the control, sword shaped leaves emerging from shoot whorl grew straight, whereas, plants treated with 0.1% colchicine showed leaf abnormalities in which emerging leaves could not grow in upward direction, but the leaves were curved and attached itself to the next growing leaves. In 0.2% colchicine concentration, leaves of treated plants showed crescent shape and in 0.3% colchicine concentration, leaves became crimpled and they first grew downward and then started to grow upward (Figure 2). 

**Figure 3:**
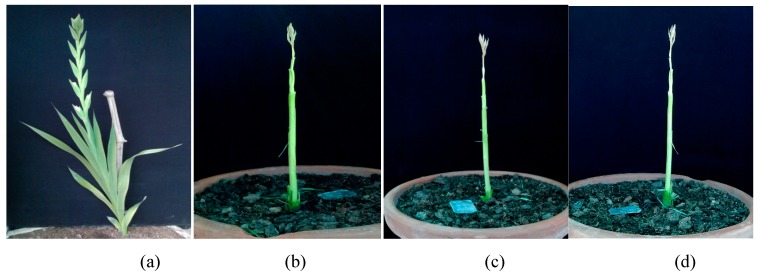
Impact of polyploidy on floral spike formation in gladiolus cv. White Prosperity: Floral spike emerged in control (**a**) however, in treated plants floral spike formation initiated but it did not elongate at 0.1% (**b**) 0.2% (**c**) and 0.3% colchicine concentration (**d**).

These types of leaf variations have been identified as mixoploids or chimeras (plant cell containing both diploid and polyploid cells). During reproductive growth, colchicine treated plants failed to blossom, but, when leaves were removed; many plants under different colchicine concentration (0.1%, 0.2% and 0.3%) initiated floral spike formation. However, these floral spikes could not further elongate as compared to control plants, where spikes emerged and they produced flowers (Figure 3). 

**Figure 4:**
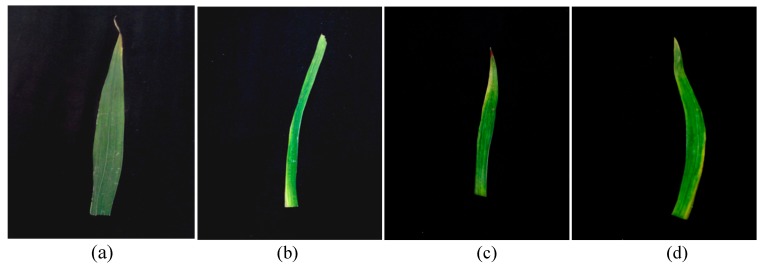
Impact of polyploidy on leaf color in gladiolus cv. White Prosperity: Leaves of dark green color have been produced in control plants (**a**) however, yellowish green (chlorine) have been developed at 0.1% (**b**), 0.2% (**c**) and 0.3% colchicine (**d**).

Along with different irregularities in plant structure, chlorophyll mutation or abnormalities like production of chlorine (Yellowish green) in mutated plants have also been observed. Gladiolus control plants had dark green color leaves but, in all colchicine treated plants, yellowish green color (chlorine) leaves were observed as shown in Figure 4.

## 5. Conclusions

During the evolution of flowering plants, a regular process known as polyploidy has a significant effect on diversity of plant species. Several advantages of polyploidy attract breeders to induce polyploidy in many ornamental crops for their improvement. Colchicine at different concentrations and time durations is used to induce polyploidy in many ornamental crops through different application methods like dipping, soaking, whole plant immersion or through cotton wool method. For successful induction of polyploidy, different direct and indirect methods have been used to identify ploidy level in plants.

## Figures and Tables

**Table 1:** Induction of polyploidy in ornamental plants by applying colchicine through dipping method since last fifteen years.

Crop	Method of Application	Most Successful Treatment	Reference
Gymnostachyum (*Gymnostachyum zeylanicum)*	Dipping of shoot cuttings in colchicine solution	Colchicine 0.1%, 24 h	Khaing et al. (2007) [39]
Orchid (*Dendrobium nobile)*	Immersing whole plant in colchicine solution	Colchicine 0.1%, 96 h	Vichiato et al. (2007) [40]
Lily (*Lilium*)	Treating bulb scales with colchicine solution	Colchicine 1%, 24 h	Balode (2008) [41]
Salvia (*Salvia coccinea* cv. Coral Nymph and cv. Lady in Red and *Salvia patens* cv. Blue Angel)	Seeds soaked in colchicine solution	Colchicine 0.05%, 24 h (*S. coccinea* cv. Coral Nymph)	Kobayashi et al. (2008) [42]
Japanese barberry (*Berberis thunbergii*)	Pre-germinated seeds immersed in colchicine solution	Colchicine 0.2%, 24 h	Lehrer et al. (2008) [33]
Jasmine tobacco (*Nicotiana alata*)	Dipping seeds in colchicine solution	Colchicine 0.5%, 24 h	El-Morsy et al. (2009) [43]
Feverfew (*Tanacetum parthenium*)	Dipping roots in colchicine solution	Colchicine 0.05% 6 h	Majdi et al. (2010) [44]
Phlox (*Phlox drummondi)*	Seed soaking method	Colchicine 0.5%, 36 h	Tiwari and Mishra (2012) [45]
Wishbone flower (*Torenia fournieri)*	Dipping of leaf petiole in colchicine solution	Colchicine 0.015% 3 d	Boonbongkarn et al. (2013) [46]
Salvia (*Salvia hains*)	Seeds dipped in colchicine aqueous solution	Colchicine 0.3 to 0.5%, 24 h	Grouh et al. (2013) [47]
Self-heal (*Prunella vulgaris* L.F. *abiflora nakai*)	Soaking of seeds in colchicine solution	Colchicine 0.05% and 0.1%, 6 h	Kwon et al. (2014) [48]
Lavandins (*Lavandula × intermedia* cvs. Grosso and Seal)	First method: Treatment of upper nodes of whole plant with colchicine Second method: Fresh cuttings completely submerged in colchicine	Colchicine 0.1%, 6 h (cutting method)	Urwin (2014) [49]
Orchid (*Dendrobium nobile*)	Plant of height 5cm was dipped in colchicine solution	Colchicine 0.1%, 96 h	Vichiato et al. (2014) [50]
Chaste tree (*Vitex agnus castus* L)	Seed soaking method	Colchicine 0.05%, 36 h	Ari et al. (2015) [12]
Calendula (*Calendula officinalis*)	Soaking of seeds in colchicine solution	Colchicine 0.08%, 4 h	El-Nashar and Ammar, (2015) [28]
Marigold (*Tagetes erecta*)	Pre-germinated seeds treated with colchicine	Colchicine 0.1%, 3-6 h: 0.2%, 3 h	He et al. (2016) [51]
Rose (*Rosa multiflora* var inermis, *Rosa roxburgii* var normalis)	First method: Germinated seeds soaked in colchicine solution Second method: Seedlings at cotyledon stage treated with colchicine	Germinating seeds method Colchicine 0.2%, 12 h	Feng et al. (2016) [52]
Matted sea-lavender (*Limonium bellidifolium*)	Seed soaking treatment	Colchicine 0.05%, 72 h	Mori et al. (2016) [53]
Swamp rosemallow (*Hibiscus moscheutos*)	Seedlings at cotyledon stage soaked in colchicine solution	Colchicine 0.1%, 24 h	Li and Ruter, (2017) [54]
White orchid tree (*Bauhinia acuminata*)	Treatment of seeds with colchicine solution	Colchicine 0.1%, 12 h (3 consecutive days	Basumatari and Das, (2017) [55]
Sultana (*Impatiens walleriana*)	Seed soaking method	Colchicine 0.05%, 48 h (2 days)	Wang and He, (2018) [56]
Gladiolus (*Gladiolus grandiflorus*)	Corm soaked in colchicine solution	Colchicine 0.1-0.3%, 24 h	Manzoor et al. (2018) [57]

**Table 2:** Induction of polyploidy in ornamental plants by applying colchicine through cotton wool method since last fifteen years.

Crop	Method of Application	Most Successful Treatment	Reference
Gladiolus wild species (*Gladiolus tristis)*	Colchicine applied to apical buds of corms through cotton wool	--------	Suzuki et al. (*2005*) [58]
Garden balsam (*Impatiens balsamina*)	Application of colchicine to seedlings through cotton plug	Colchicine 0.4%, 3 d	Anurita and Girjesh (2007) [59]
African violets (*Saintpaulia ionantha*)	Leaf petiole treated by cotton-based method	All colchicine concentration (0.04%, 0.06% and 0.09%), 22.5-23.5 h	Seneviratine and Wijesundra (2007) [60]
Petunia (*Petunia hybrida*)	Shoot apics of seedlings were treated with colchicine through cotton wool	Colchicine 0.2%, 48 h	Ning et al. (2009) [61]
Crape myrtle (*Lagerstroemia indica* cvs. Zi Wei, Hong Wei and Yin Wei)	Colchicine applied to apical meristerm of young seedlings through cotton wool	Colchicine 0.5%, 72 h (cv. Zi Wei); 0.8%, 48 h (cv. Hong Wei.); 0.2%, 96 h; 0.5%, 48 h; 0.8%, 72 h (cv. Yin Wei.)	Ye et al. (2010) [62]
African marigold (*Tagates erecta*)	First method: Placing of whole plant with roots in a colchicine solution Second method: Treatment of apical buds with colchicine-soaked cotton	Colchicine 0.0005%, 6 h	Sadhukhan et al. (2014) [63]
Chrysanthemum (*Dendranthema indicum* var. *aromaticum)*	First method: Grin seeds soaked in colchicine solution Second method: Pre-germinated seeds dipped in colchicine aqueous solution Third method: Cotton balls placed on shoot tips; colchicine poured on cotton balls through micropipette	Colchicine 0.1%, 24 h (grin seeds); 0.1%, 7 d (shoot tips)	He et al. (2016) [64]
Bougainvillea (Bougainvillea *spp* cvs Lalbagh, Mahara)	Colchicine soaked cotton placed on dormant nodal buds	Colchicine 0.4% for 72 h (cv. Lalbagh); 0.3% for 72 h (cv. Mahara)	Anitha et al. (2017) [65]
Chrysanthemum (*Chrysanthemum carinatum*)	Apical buds treated with colchicine-soaked cotton swab	Colchicine 0.2 %, 3 days with 6 h duration per day	Kushwah et al. (2018) [66]

**Table 3:** Induction of polyploidy in ornamental plants by applying colchicine with semi solid agar method since last fifteen years.

Crop	Method of Application	Most Successful Treatment	Reference
Balloon flower (*Platycodon grandiflorus*)	Warm semi solid (1% agar) colchicine applied to the apical buds of the seedlings	Colchicine 0.5%, 72 h	Wu et al. (2011) [67]
Balloon flower (*Platycodon grandiflorus*)	Treatment of apical buds with colchicine	Colchicine 0.5%, 72 h	Wu et al. (2012) [68]
Clematis (*Clematis heracleifolia*)	Apical buds treated with warm semi solid colchicine (1% agar)	Colchicine 0.2%, 48 h	Wu et al. (2013) [69]
Japanese privet (*Ligustrum japonicum*)	Newly growing points treated with colchicine stock (0.55% semi solid agar)	Colchicine 0.2%, three consecutive days	Fetouh et al. (2016) [70]

**Table 4:** Induction of polyploidy in ornamental plants by applying colchicine through dropping method since last fifteen years.

Crop	Method of Application	Most Successful Treatment	Reference
London plane (*Planatus acerifolius*)	First method: Seeds soaked in colchicine solution Second method: Apical meristems of seedling treated with colchicine solution through dropping method	Colchicine 0.3%-0.4%, 24 h	Liu et al. (2007) [71]
Pelargonium (*Pelargonium graveolens* cvs. Black Velvet Scarlet F1 and Gizela F1)	Colchicine applied on seedling apics at true leaf stage	Colchicine 0.5%, 3 d (cv Black Velvet Scarlet F1); 1.0%, 2 d; 2.5%, 2 d; 0.5%, 5 d (cv. Gizela F1)	Jadrna et al. (2010) [72]
Garden balsam (*Impatiens balsamina sp Xinhua impatiens* and *Camellia impatiens*)	Application of colchicine to shoot apics of seedlings	Colchicine 0.5%, 96 h (*Xinhua impatiens*); 0.5%, 72 h (*Camellia impatiens*)	Xiaohua et al. (2011) [73]
Madagascar periwinkle (*Catharanthus roseus*)	Seedling shoot apics treatment with colchicine	Colchicine 0.4%, 7 d	Hosseini et al. (2013) [74]
Zinnia (*Zinnia violacea* cv. Oklahoma White and *Zinnia angustifolia* cv. Crystal Orange)	Colchicine applied drop wise to growing points	Colchicine 0.33%	Gu (2015) [75]
Anise hyssop (*Agastache foeniculum*)	Apical meristems treated drop wise with colchicine	Colchicine 0.006% for three successive days	Talebi et al. (2017) [76]
Moth orchid (*Phalaenopsis pulcherrima*)	Treatment of seeds with colchicine through dropping method	Colchicine, 0.5%	Soetopo and Hosnia. (2018) [77]
Chrysanthemum (*Dendranthema grandiflorus*)	Cotton covered shoots treated drop wise with colchicine	Colchicine, 0.8% for 6 days	Lertsutthichawan et al. (2018) [78]
